# Comparison of laser versus cold knife visual internal urethrotomy in the treatment of urethral stricture (stricture length <2 cm): A systematic review and meta-analysis

**DOI:** 10.1097/MD.0000000000037524

**Published:** 2024-05-03

**Authors:** CaiXia Chen, Jiao Qin, ChongJian Wang, HaoTian Huang, HongYuan Li, Zhi Wen, Yang Liu, XueSong Yang

**Affiliations:** a Department of Urology, Affiliated Hospital of North Sichuan Medical College, Nanchong, China.

**Keywords:** analysis safety, cold knife visual internal urethrotomy, laser urethrotomy, meta, urethral stricture

## Abstract

**Purpose:**

There is still controversy regarding the safety and efficacy of cold knife visual internal urethrotomy and laser incisions for the treatment of urethral stricture. This study aims to compare the results of postoperative long-term and short-term maximum urinary flow rates (Qmax), surgical time, postoperative complications, and 1-year recurrence rates between the cold knife and laser surgery.

**Methods:**

We searched databases including Embase, PubMed, Cochrane, and Clinical Trials.gov to identify relevant literature published in English up to September 2023. We used Stata to compare various parameters. This study is registered in PROSPERO (CRD42023471634). Nine comparative experiments were conducted, involving a total of 659 participants.

**Results:**

The laser group showed significantly better results compared to the cold knife group in terms of postoperative 12-month maximum urinary flow rate (mean differences [MD] 2.131; 95% [1.015, 3.249], *P* < .0001), postoperative bleeding (RR 0.277, 95% [0.079, 0.977], *P* = .046), and 1-year recurrence rate (RR 0.667, 95% [0.456, 0.976], *P* = .037). However, there were no significant differences in postoperative 6-month and 3-month Qmax, surgical time, urethral leakage complications, overall complications, and Visual Analog Scale (VAS) scores.

**Conclusion:**

The current study results suggest that laser urethral incision has greater advantages in the long-term (12 months), 1-year recurrence rate, and bleeding complications compared to cold knife urethral incision in the treatment of urethral stricture (<2 cm). Therefore, laser urethral incision may be a better choice for patients with urethral stricture.

## 1. Introduction

Urethral stricture is caused by ischemic fibrosis of the corpora spongiosa,^[1]^ resulting in narrowing of the urethral lumen, which in turn leads to difficulty with urination and has a significant adverse impact on the physical and psychological well-being of patients.^[2]^ Common risk factors for urethral stricture in men include urethral infections, a history of trauma, prior endoscopic procedures, urethral catheterization, or mechanical implantation procedures. However, the etiology of urethral stricture in many men is idiopathic.^[3,4]^ An idiopathic urethral stricture occurs in 34% and 63% of cases at the penile and bulbar regions, respectively.^[4]^ Urethral stricture is a condition associated with a high risk of recurrence, with Pansadoro and Emiliozzi reporting a recurrence rate of up to 58% for bulbar urethral strictures after direct vision cold knife urethrotomy.^[5]^ This high recurrence risk is attributed to the inability of urethral dilation, urethral reconstruction, or urethrotomy to completely remove urethral scar tissue and inhibit scar growth.

Direct Visual Internal Urethrotomy (DVIU) was first performed by Ravasini in 1957.^[6]^ He described the use of an electric knife to incise narrow urethral strictures, which inevitably resulted in significant thermal effects on the surrounding healthy tissues. Subsequently, in 1971, Sachse introduced the urethrotome, allowing surgeons to perform urethral incisions using a cold knife, with reported success rates as high as 80%. Compared to the earlier use of the electric knife, cold knife urethrotomy avoided the thermal effects on surrounding healthy tissues.^[7]^ DVIU gained popularity among urologists due to its simplicity, speed, and short recovery period.^[6,8,9]^ Later, laser urethrotomy was introduced in 1979. However, it did not initially receive sufficient attention from surgeons.^[10]^ It was only later when holmium: YAG laser urethrotomy showed results similar to cold knife incisions in treating urethral strictures <1.5 cm, that it gained attention.^[11,12]^ Laser urethrotomy offers efficient energy ablation and excellent hemostasis, providing a clear view during the surgical procedure. Additionally, due to the laser shallow penetration depth of only 0.4 cm, it minimizes damage to surrounding normal structures.^[13,14]^ However, laser urethrotomy requires high equipment and technical demands. Currently, according to guidelines, DVIU remains the first-line treatment for bulbar urethral strictures.^[1]^

Laser and cold knife are the 2 most commonly used methods for internal urethrotomy, but their efficacy and safety have been a subject of ongoing debate.^[15,16]^ Zheng^[16]^ conducted a meta-analysis systematic review to study the superiority of these 2 treatment methods. However, their study has some limitations, including the inclusion of only 7 articles with a total of 453 patients, and the absence of stratified analysis based on stricture location and length. Therefore, our study aims to integrate existing clinical research, incorporating the latest literature, with the hope of addressing the limitations of previous studies and providing clinicians with the most up-to-date evidence for clinical decision-making.

## 2. Methods

This meta-analysis was conducted following the Preferred Reporting Items for Systematic Reviews and Meta-Analyses^[17]^ Statement, and it is registered in PROSPERO under the registration number CRD42023471634.

### 2.1. Literature search strategy

We conducted a comprehensive search for relevant literature with a cutoff date in September 2023, using Embase, PubMed, Cochrane, and ClinicalTrials.gov. We utilized the following MeSH terms and keywords for our search: “urethral stricture” “Laser Therapy” and “cold knife visual internal urethrotomy.” In addition, we manually searched and reviewed relevant literature to avoid any omissions, and the references included only studies reported in English.

### 2.2. Inclusion and exclusion criteria

We formulated inclusion criteria using the PICOS (Patient, Intervention, Comparison, Outcome, Study Type) method. P) Patients clinically diagnosed with urethral stricture <2 cm in length and older than 18 years; I) Experimental group patients undergoing laser urethrotomy; C) Control group patients undergoing cold knife urethrotomy; O) One or more of the following outcomes: maximum urine flow rate, surgical duration, postoperative complications, 1-year recurrence rate; S) Prospective studies or randomized controlled trials (RCTs). The exclusion criteria are as follows: non-comparative studies; editorial reviews, conference abstracts, case reports, unpublished studies, or reviews; studies for which analytical data cannot be obtained.

### 2.3. Study screening and selection

The data for our study were independently extracted by 2 researchers (CCX and QJ). Initially, a review of the titles and abstracts of the retrieved articles was conducted, and articles meeting the inclusion criteria underwent a full-text assessment. Data extraction and analysis were carried out independently by 2 researchers, and in case of any discrepancies, a third researcher (WCJ) was consulted to resolve them.

### 2.4. Data items

We extracted the following data from articles that met the inclusion criteria: the type of included studies (RCT or other types), intervention measures, total number of patients, age, follow-up duration, length of urethral stricture, etiology of stricture, stricture location, surgical duration, maximum urine flow rate (Qmax) at different postoperative time points, postoperative complication rates, and postoperative recurrence rates. Any discrepancies were resolved through consensus or consultation with a third researcher.

### 2.5. Bias risk assessment

We used the Cochrane risk of bias assessment tool to evaluate potential bias in randomized controlled trials (RCTs). This tool examines 5 domains in RCTs: the randomization process (selection bias), deviations from intended interventions (performance bias), incomplete outcome data (attrition bias), measurement of the outcome (detection bias), and selection of the reported result (reporting bias). These domains are categorized as low risk, unclear risk, and high risk.^[18]^ For non-RCT studies, we employed the Newcastle-Ottawa Scale (NOS) to measure the risk of bias. Using a semi-quantitative star system to assess the quality of the literature, a total of 8 stars were available, and studies with a score of 6 stars or more were included.

### 2.6. Statistical analysis

In this study, we performed statistical analysis using Stata V17 software. The results are reported with 95% confidence intervals (CIs) and relative risks (RR) for binary variables and mean differences (MD) for continuous variables. For studies that provided only the median, quartiles, or range, data were transformed into mean (MD) and standard deviation (SD).^[19,20]^ We assessed study heterogeneity using the *I*^2^ statistic, with *I*^2^ < 50% indicating low heterogeneity and analyzed using a fixed-effects model, while *I*^2^ > 50% indicated substantial heterogeneity and analyzed using a random-effects model. Interpretations are as follows: 0% to 40% represents low heterogeneity; 40% to 60% indicates moderate heterogeneity; 60% to 90% signifies substantial heterogeneity; 90% to 100% indicates considerable heterogeneity.^[21]^ *I*^2^, along with the Cochran Q test, was used to quantify the heterogeneity of the studies.^[21]^ *P* value < .05 was considered statistically significant.

### 2.7. Sensitivity analysis

To ensure the stability of our results, we conducted a sensitivity analysis using Stata software and employed a leave-one-out method to detect heterogeneity in the included literature. This method involves systematically excluding 1 study at a time from the overall effect estimate. Additionally, we examined robustness by considering the size of the cohort studies, which could be a source of heterogeneity. However, it should be noted that sensitivity analysis cannot be performed when comparing 3 or fewer studies.

### 2.8. Publication bias

When the number of included studies is <10, the power of the tests is insufficient; therefore, we did not perform publication bias analysis.^[22]^

## 3. Result

### 3.1. Baseline characteristics

**Figure 1.**
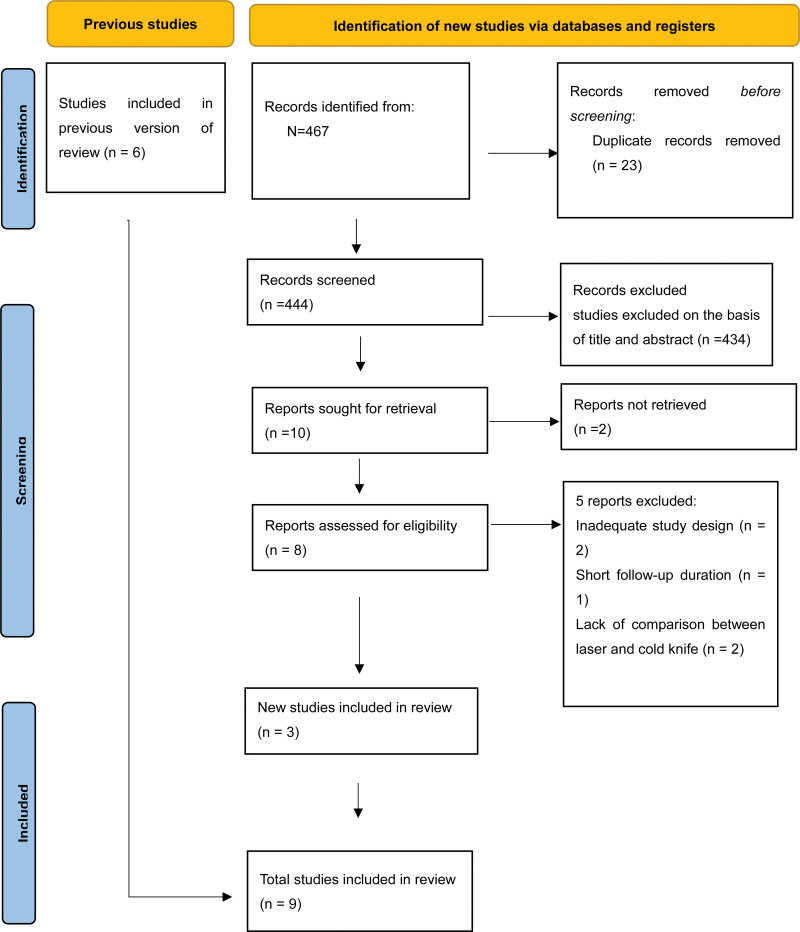
RPISMA flowchart of study selection.

Figure 1 shows that we initially identified 467 articles, and after removing duplicates, 444 remained. We excluded 434 studies after reviewing the titles and abstracts, and a further 7 articles were excluded after reading and screening the full texts. Three newly included studies, in addition to the 6 studies from the previous meta-analysis, resulted in a total of 9 studies (4 randomized controlled trials and 5 non-randomized controlled trials) involving 659 patients included in our study. These 9 studies were published between 2010 and 2023 (Table 1).^[6,8–10,14,23–25]^ Among them, 4 studies were RCTs,^[6,8,9,14]^ and the other 5 studies^[10,23–25]^ were prospective. The follow-up duration varied from 3 months to 1 year. In all studies, the patients were adults, and there were no baseline data differences between patients in each study (Table 2). The causes of stricture included traumatic, iatrogenic, infectious, and idiopathic. The location of the strictures varied, with the average length of stricture being <2 cm in all studies.

**Table 1:** Demographic characteristics of the included studies.

Study	Design	Size		Follow-up (mo)	Age(yr)		Stricture length		Etiology of stricture							
		Group L	Group C		Group L	Group C	Group L	Group C	Group L	Group C	Group L	Group C	Group L	Group C	Group L	Group C
									Traumatic		Inflammatory		Iatrogenic		Idiopathic	
Maged2023	RCT	33	33	12	42.58 (9.32)	44.23 (12.04)	1.68 (0.51)	1.23 (0.38)	6	5	2	2	22	24	3	2
Gamal 2021	RCT	40	40	12	46.68 (13.19)	47.58 (13.26)	<1 cm	<1 cm	7	7	11	11	19	16	3	6
Mohamed2022	Prospective	30	30	6	44 (10.59)	43.00 (11)	<1.5 cm	<1.5 cm	4	5	7	10	15	13	4	2
Mustafa2018	Prospective	34	29	12	55.3 (8.9)	54.80 (9.5)	<2 cm	<2 cm	5	7	2	4	21	21	1	2
Sudhir2014	Prospective	45	45	12	NA	NA	<2 cm	<2 cm	2	8	42	19	16	13	4	5
Slawomir2012	Prospective	25	25	6	61.2 (16.1)	65.70 (11.9)	1.86 (1.26)	1.66 (1.02)	NA							
Mustafa2011	RCT	21	30	12	63.85 (7.98)	59.70 (15.29)	1.109 (3.28)	1.23 (2.98)	NA							
Zbigniew2010	RCT	30	20	12	66.9 (8.33)	57.60 (16.32)	1.23 (0.39)	1.64 (0.56)	4	1	0	1	17	13	9	5
Ankur2016	Prospective	52	55	6	38.13 (12.3)	39.38 (13.4)	1.34 (0.25)	1.31 (0.25)	NA							

Group c = group cold knife, group L = group laser, NA = not available, RCT = randomized controlled trial.

**Table 2:** The demographics of the studies.

Variable	Number of studies with available data	WMD/RR	95% CI	*P*-value
Age (yr)	8	0.65	−1.9,3.19	.647
Stricture length (cm)	5	0.06	−0.26,0.37	.774

Cl = confidence interval, RR = risk ratio, WMD = weighted mean difference

### 3.2. Assessment of quality

**Figure 2.**
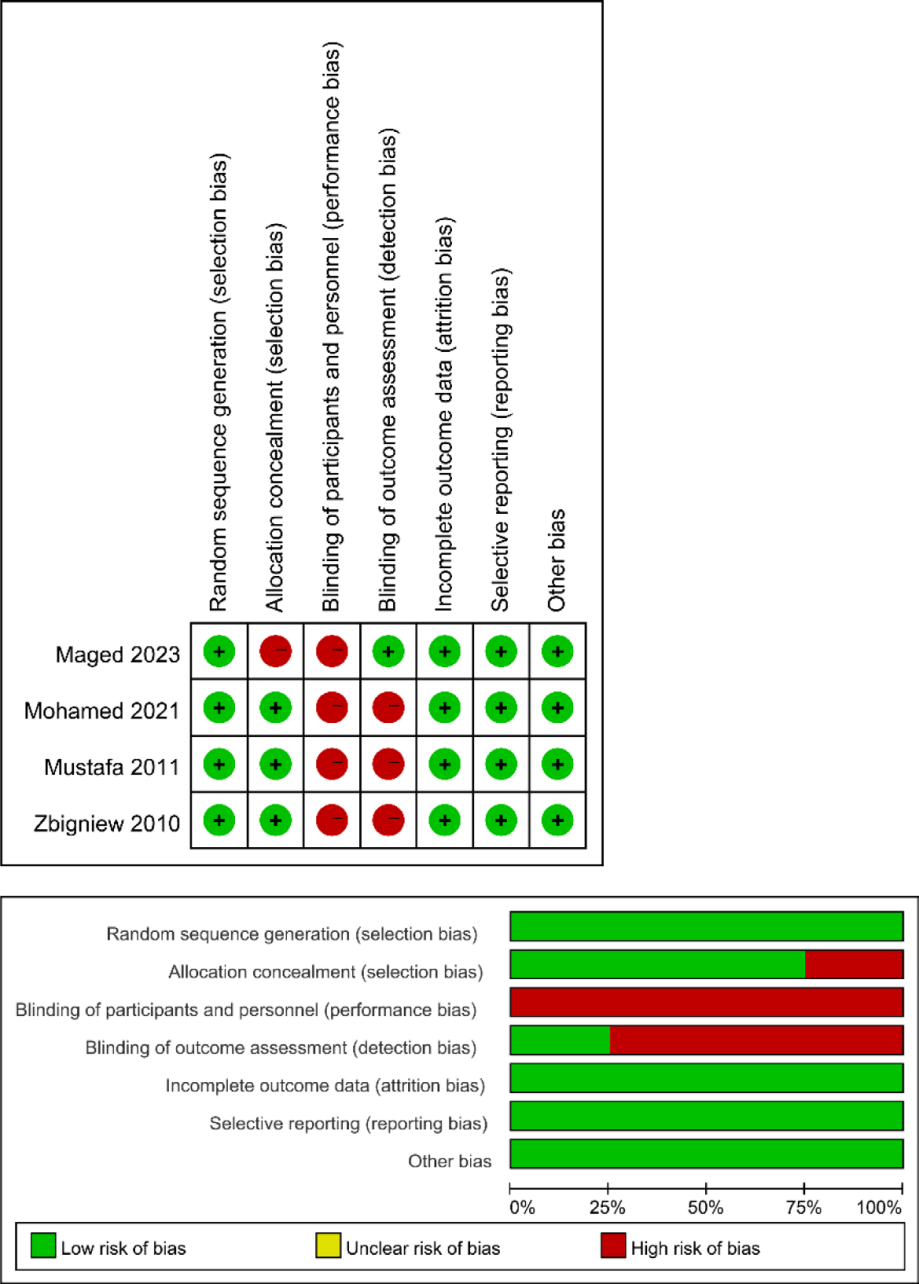
Quality assessment of randomized controlled studies.

A score of 6 stars or higher on the NOS was defined as high-quality literature. All 5 included prospective studies scored 6 stars or higher in the NOS, indicating good quality. Among the 4 randomized controlled trials, 4 of them did not perform blinding of participants and personnel (performance bias), and 3 of them did not do blinding of outcome assessment (detection bias), while Maged et al did not do allocation concealment (selection bias), making them at high risk of bias.^[18]^ The quality assessment of randomized controlled trials is detailed in Figure 2, and the assessment of prospective studies is detailed in Table 3.

**Table 3:** Study quality of case-control studies based on the Newcastle-Ottawa scale.

NOS		Selection				Comparability	Exposure			Total score
		1	2	3	4	5	6	7	8	
Mohamed 2022	Prospective	★	★	★	★	★	★	★	★	8/High
Mustafa 2018	Prospective		★	★	★	★		★	★	7/High
Sudhir 2014	Prospective		★	★	★	★	★	★	★	7/High
Slawomir 2012	Prospective		★	★	★	★	★	★	★	7/High
Ankur 2016	Prospective		★	★	★	★	★		★	6/High

1: Representativeness of the exposed cohort; 2: Selection of the nonexposed cohort; 3: Assessment of exposure; 4: Demonstration that outcome of interest was not present at the start of the study; 5: Comparability of cohorts based on the design or analysis; 6: Ascertainment of outcome; 7: Long enough follow-up for outcomes to occur; 8: Adequacy of follow-up of cohorts.

NOS = Newcastle-Ottawa Scale.

### 3.3. Outcome analysis

#### 3.3.1. Qmax.

**Figure 3.**
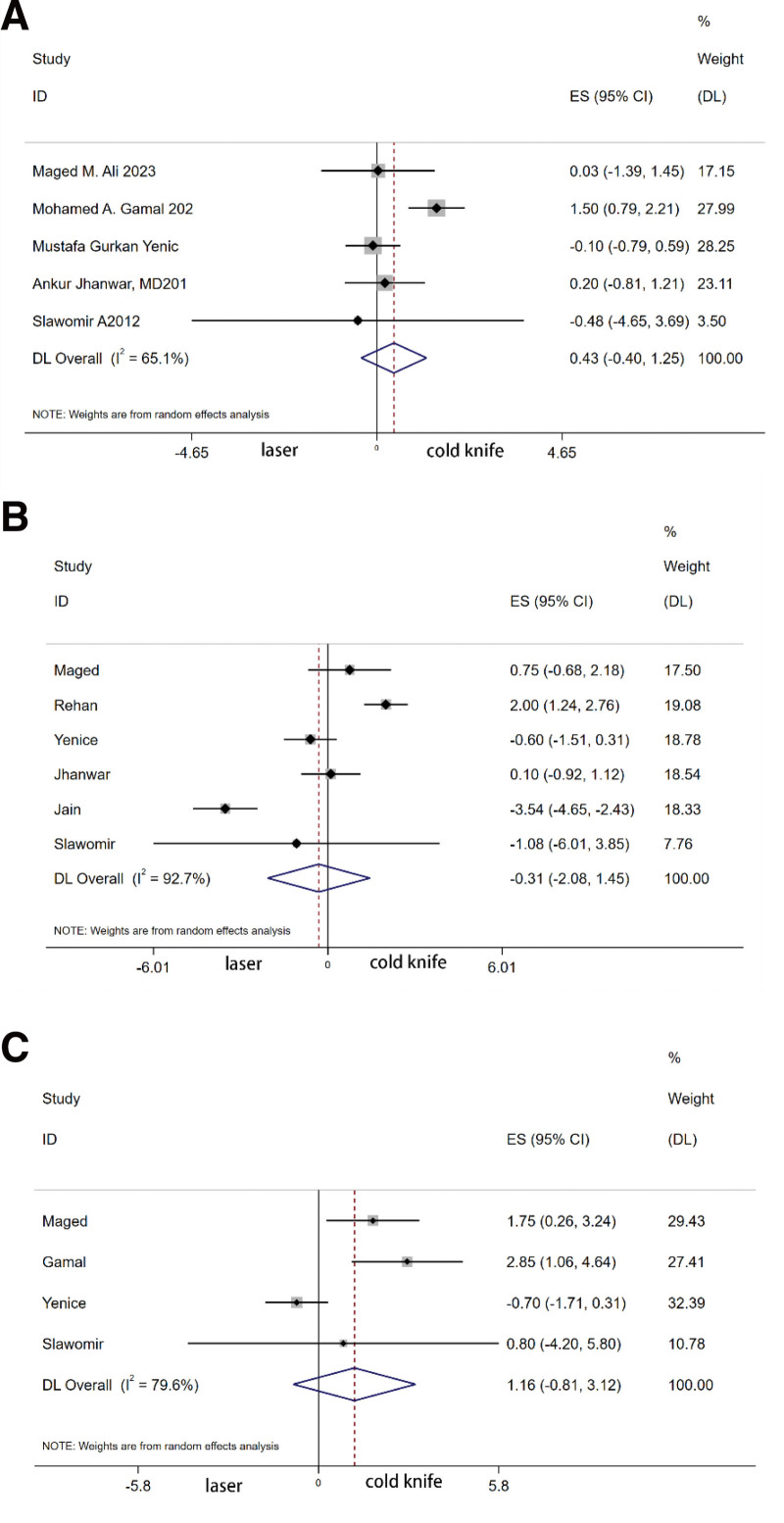
Forest plot of maximum urinary flow (Qmax) after laser urethrotomy and after cold knife urethrotomy (A) Qmax (3 mo) (B) Qmax (6 mo) (C) Qmax (12 mo).

The summary results of 5 studies showed that there was no significant difference in the maximum urine flow rate at 3 months postoperatively between the laser group and the cold knife group (MD 0.426, 95% CI [−0.395, 1.248, *P* = .345]); the summary analysis of 6 studies revealed no significant difference in the maximum urine flow rate at 6 months postoperatively between the laser group and the cold knife group (MD −0.314, 95% [−2.077, 1.449], *P* = .732); the summary results of 3 studies showed that the maximum urine flow rate at 12 months was significantly better in the laser group than in the cold knife group (MD 2.131, 95% [1.015, 3.249], *P* < .0001) (Fig. 3).

### 3.4. Surgical time

**Figure 4.**
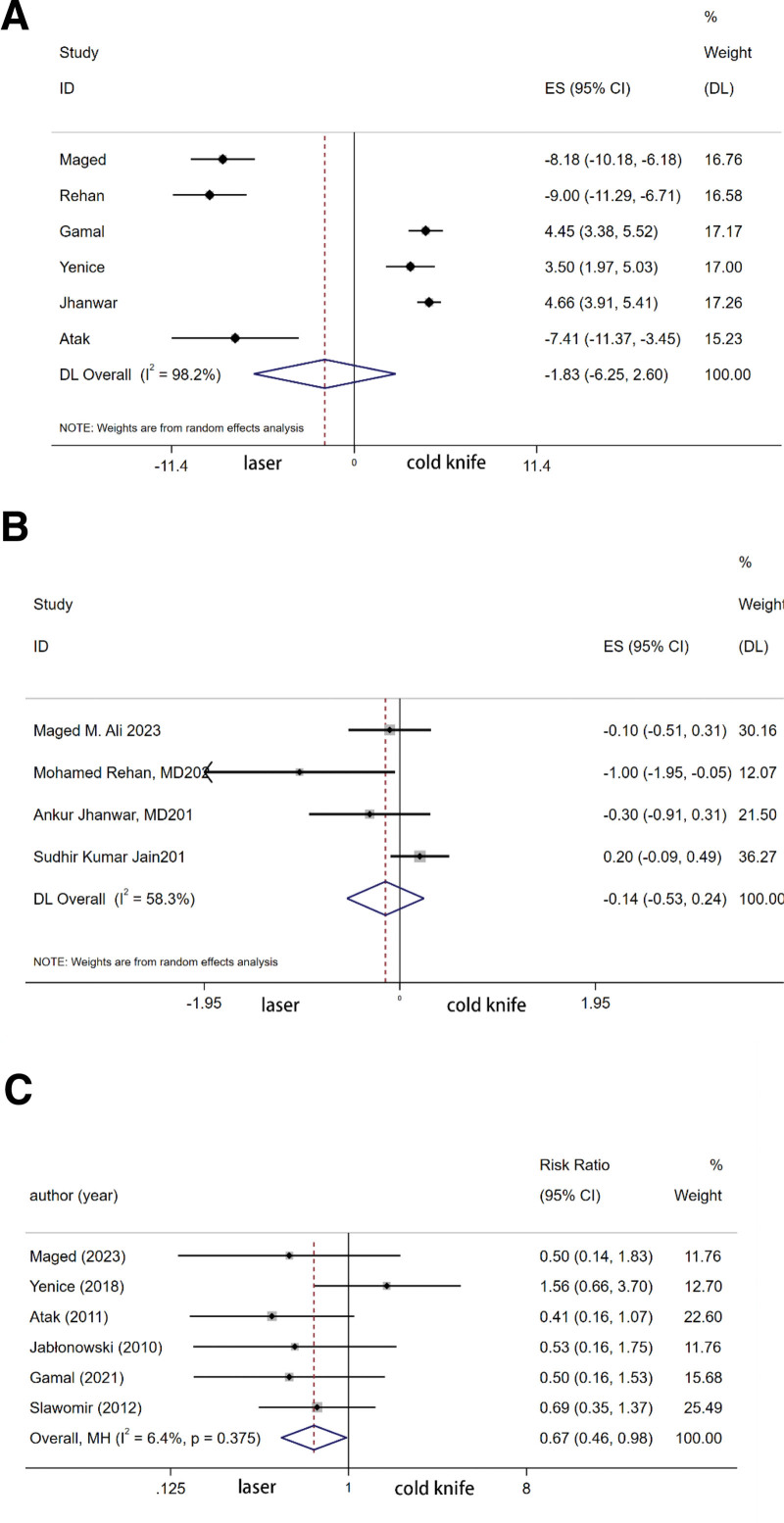
Forest plot of (A) operation time (B) VAS pain score (C) Recurrence (1 yr). VAS = Visual Analog Scale.

The summary analysis of 8 studies showed that there was no significant difference in surgical duration between the laser group and the cold knife group (ES −1.828, 95% [−6.251, 2.596], *P* = .529) (Fig. 4).

### 3.5. Complications

**Figure 5.**
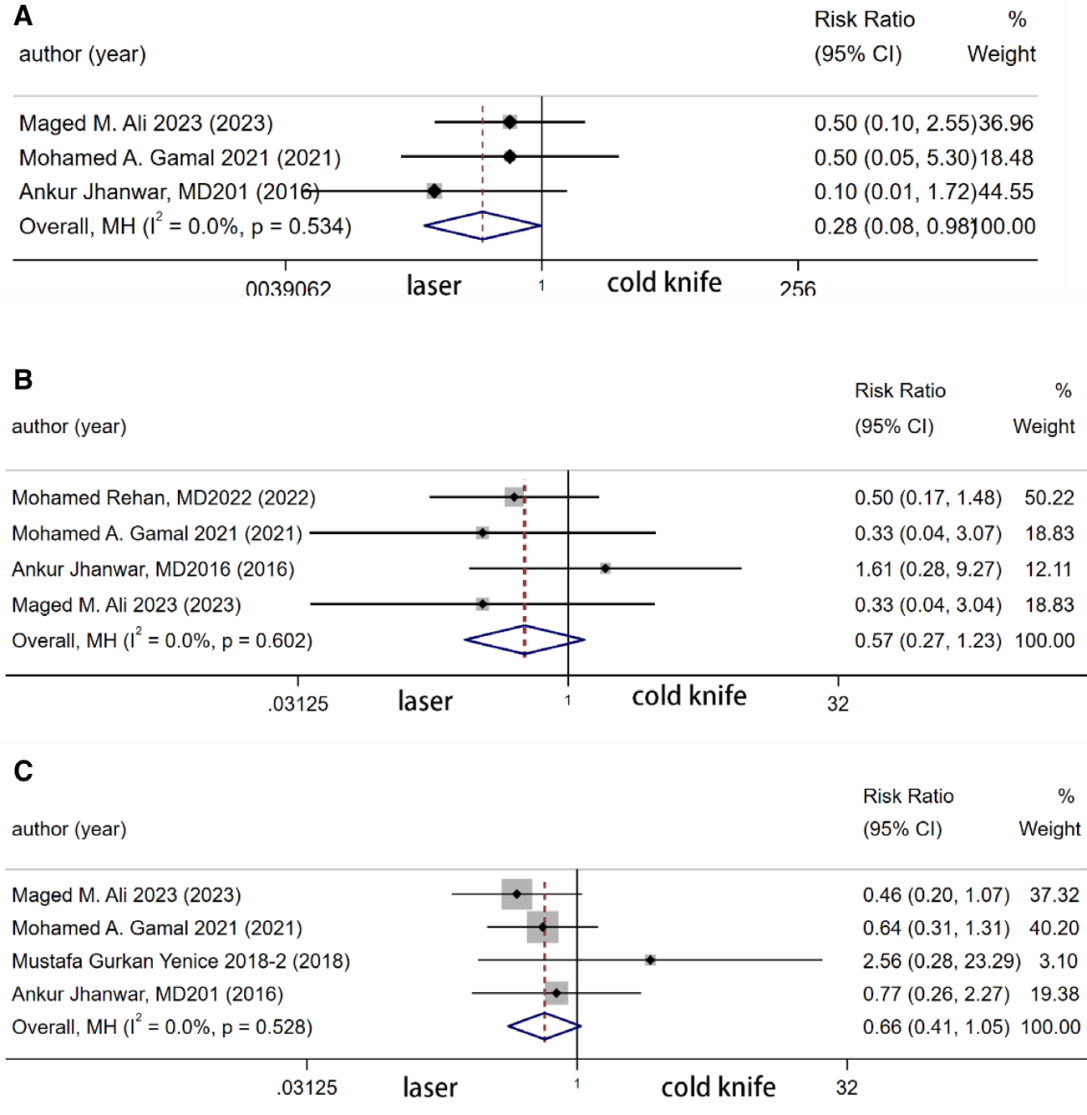
Forest plot of complication (A) bleeding per urethra (B) fluid extravasation (C) general complication.

The summary analysis of 3 studies showed that the laser group had a significantly lower rate of bleeding compared to the cold knife group (RR 0.277, 95% [0.079, 0.977], *P* = .046). The summary analysis of 4 studies revealed no significant difference in the occurrence of urethral stricture complications between the 2 groups (RR 0.572, 95% [0.265, 1.233], *P* = .154). The summary analysis of 4 studies indicated no significant difference in overall complications between the 2 groups (RR 0.659, 95% [0.413, 1.051], *P* = .08) (Fig. 5).

#### 3.5.1. 1-year recurrence rate.

The summary analysis of 5 studies demonstrated that the 1-year recurrence rate after laser surgery was significantly lower than after cold knife surgery (RR 0.667, 95% [0.456, 0.976], *P* = .037) (Fig. 4).

#### 3.5.2. Visual Analog Scale (VAS) pain score.

The summary analysis of 4 studies showed that there was no significant difference in postoperative VAS pain scores between the 2 surgical methods (ES −0.143, 95% CI [−0.528, 0.242], *P* = .554) (Fig. 4).

### 3.6. Sensibility analysis

Most of the outcomes had moderate to low heterogeneity, but the postoperative maximum urine flow rate and surgical duration outcome measures exhibited high heterogeneity. For postoperative maximum urine flow rate (3-month group), the heterogeneity test resulted in *I*^2^ = 65.09%, indicating significant heterogeneity. For the postoperative urine flow rate (6-month group), the heterogeneity test resulted in *I*^2^ = 92.7%, indicating substantial heterogeneity. For the postoperative urine flow rate (12-month group), the heterogeneity test resulted in *I*^2^ = 79.6%, indicating significant heterogeneity. For surgical duration, the heterogeneity test resulted in *I*^2^ = 97.8%. For the VAS pain score, the heterogeneity test resulted in *I*^2^ = 58.3%, indicating moderate heterogeneity. *I*^2^ > 50% for these outcome measures; therefore, a random-effects model was used for the meta-analysis. Interpretation of these results must be cautious, and more studies are needed for confirmation. However, the low to moderate consistency of these results may also introduce bias, as *I*^2^ exhibits substantial variability in a small number of studies.^[26]^

## 4. Discussion

Currently, DVIU is the first-line treatment for urethral stricture, with a cure rate of up to 90%.^[1]^ DVIU is favored by urologists due to its simplicity, speed, and shorter recovery period.^[6,8,9]^ Urethral stricture is a condition with a potentially high recurrence risk because regardless of urethral dilation, urethral reconstruction, or urethrotomy, it is impossible to avoid the complete removal of urethral scar tissue and the inhibition of scar growth. Laser treatment, on the other hand, works based on vaporization. The Holmium laser, with its high wavelength of 2140 nm and a short emission time of 0.25 mL/s, offers an effective option for managing urethral strictures by vaporizing fibrotic scar tissue with minimal thermal damage to normal tissues.^[27]^ Different types of lasers include carbon dioxide, argon, diode, excimer, Nd: YAG (neodymium-doped yttrium aluminum garnet), KTP (potassium titanyl phosphate), holmium-doped yttrium aluminum garnet, thulium laser, and more. However, the current literature has not mentioned the superiority of 1 laser type over another.^[3]^ In a study by Mohamed,^[23]^ the thulium laser group was compared to the cold knife group, with a recurrence rate of 13.3% in the laser group and 25% in the cold knife group (*P* = .01). Jabłonowski and colleagues^[8]^ used a neodymium-doped yttrium aluminum garnet laser in the laser group. They observed a 1-year recurrence rate of 20% in the cold knife group and 6.67% in the laser group (*P* = .013). However, current research results suggest that laser treatment is superior to cold knife treatment.

### 4.1. Surgical time

In this study, through the meta-analysis of 8 studies, we concluded that there is no significant difference between the 2 surgical methods in terms of surgical duration. This result is inconsistent with the previous study by Zheng, which included only 4 studies, while our study included 8 articles, including 3 recent ones. Maged study showed that the average surgical duration in the laser group was 18.11 ± 3.92 minutes, while in the cold knife group, it was 26.29 ± 4.34 minutes, with a significant difference (*P* < .001).^[6]^ However, the length of urethral stricture in the laser group (1.68 ± 0.51) was shorter than that in the cold knife group (1.23 ± 0.38). Additionally, Mustafa study showed that the average surgical time in the laser group was 16.42 ± 8.04 minutes, while in the cold knife group, it was 23.83 ± 5.47 minutes, with a significant difference (*P* < .001). However, the length of urethral stricture in the laser group (1.109 ± 3.28) was longer than that in the cold knife group (1.23 ± 2.98).^[9]^ Therefore, a cross-sectional comparison of the length of urethral stricture did not significantly affect the surgical duration. However, some research results suggest that the surgical time used in the cold knife group is significantly shorter than that in the laser group.^[10,14,24,28]^ This may be related to the greater technical difficulty of using lasers for urethral incisions and the lack of experience among surgeons.^[28]^ However, these issues are expected to be resolved with the wider adoption of laser technology and the accumulation of surgical experience. Regarding surgical duration, the heterogeneity test showed *I*^2^ = 98.9%, indicating strong heterogeneity among the selected literature in this study. After conducting sensitivity analysis using the leave-one-out method, it was found that the heterogeneity was mainly attributed to the study by Jain. After excluding this study and performing the meta-analysis again, the heterogeneity test showed *I*^2^ = 98.24%, indicating that the results are more robust. Considering the outcome measures of the studies and the included articles, it was revealed that the primary reasons for heterogeneity in surgical duration among different articles are the varying proficiency of surgeons in the surgical method and the differences among different surgeons. Therefore, this heterogeneity cannot be resolved through subgroup analysis or other methods, and high-quality, multicenter studies are still needed for validation

### 4.2. Complications

We observed that the incidence of postoperative bleeding complications in the laser group was significantly lower than that in the cold knife group, which is consistent with Zheng results. This was expected because the depth of laser penetration is only 0.4 cm, resulting in less damage to surrounding normal tissues, and the laser also has effective hemostatic capabilities. The incidence of bleeding complications in the laser group was 2.3% (3 out of 127 cases), while it was 8.4% in the cold knife group (11 out of 131 cases). The most commonly accepted method to reduce bleeding is to make incisions at the 12 o’clock position of the corpus spongiosum or in areas with more extensive scar tissue. However, Kamp and colleagues compared using a holmium laser for incisions at the 12 o’clock and 6 o’clock positions and found no significant differences in bleeding rates and other complications between the groups.

There was no significant difference in the incidence of other complications between the 2 groups. The incidence of urethral fistula complications in the laser group was 5.7% (9 out of 157 cases), while it was 10% in the cold knife group (16 out of 161 cases). Meta-analysis of the incidence of urethral fistula complications showed no significant difference between the 2 groups (RR 0.572, 95% CI [0.265, 1.233], *P* = .154). When performing urethral incision with laser surgery, due to a lack of depth perception, surgeons may cause inadequate or excessive cutting, leading to early complications such as urethral fistula. Therefore, Jain study recommended a lower frequency, 7 Hz, to minimize penetration and coagulation of deeper tissues in the urethra. Moreover, when passing the laser fiber through the narrow urethra, it may damage the urethra, so laser intraurethral incision requires higher technical proficiency among surgical personnel, which might lead to a corresponding increase in surgical duration.

The overall complication rate in the laser group was 14.2% (23 out of 161 cases), while it was 21.9% in the cold knife group (35 out of 160 cases). Meta-analysis of the overall complication rate showed no significant difference between the 2 groups (RR 0.659, 95% CI [0.413, 1.051], *P* = .08).

The incidence of postoperative urinary tract infection complications in the laser group was 6.8% (5 out of 73 cases), the same as in the cold knife group (5 out of 73 cases), which includes 2 studies. The incidence of postoperative fever complications in the laser group was 3.4% (3 out of 87 cases), while it was 2.2% in the cold knife group (2 out of 91 cases). Since there were only 2 articles for postoperative urinary tract infection and postoperative fever complications, no combined meta-analysis was conducted, and we look forward to more high-quality, multicenter studies for verification.

### 4.3. One-year recurrence rate

In this study, a meta-analysis of 5 studies was conducted, and the conclusion was that the 1-year recurrence rate in the laser group (18.6%) was significantly lower than that in the cold knife group (28.5%). The cornerstone of preventing urethral stricture recurrence is the removal of all scar tissue and fibrosis. Traditional cold knife incision surgery cannot remove fibrotic tissue and cannot inhibit scar tissue from regrowing. However, surgeons has found relief in holmium laser, which vaporizes a substantial portion of scarred tissues without affecting healthy tissues due to its shallow penetration depth of only 0.4 mm. This makes the 1-year recurrence rate significantly lower in the laser group compared to the cold knife group.

At the 12-month follow-up, Cecen^[29]^ and Ozcan^[30]^ considered that patients who did not complain of voiding difficulties and had a maximum urine flow rate > 12 mL/s were considered free from recurrence. On the other hand, Atak^[9]^ and Jhanwar^[24]^ considered patients without voiding complaints and a maximum urine flow rate > 10 mL/s as not having recurrence after the surgery. Tam and colleagues reported that both the maximum urine flow rate and IPSS testing had low sensitivity in detecting urethral stricture recurrence.^[31,32]^

## 5. Limitations

This study has some limitations that need to be considered. Firstly, the number of included randomized controlled trials (RCTs) and the sample size are relatively small (only 4 RCTs and 5 prospective controlled trials, totaling 659 patients). Additionally, publication bias was not assessed. Secondly, our study found a correlation between the recurrence rate of urethral stricture and the length of the stricture. For each 1 cm increase in stricture length, the risk of recurrence increased by 1.22.^[16]^ However, the studies we selected did not evaluate whether stricture length could independently predict the success rate or classify outcomes based on stricture length. Similarly, the selected studies reported different stricture locations, making it difficult to analyze the correlation between stricture location and postoperative parameters. Thirdly, there is no standardized indicator for postoperative urethral stricture. Follow-up for urethral stricture recurrence requires highly sensitive and specific indicators. Therefore, more sensitive postoperative follow-up indicators and further high-quality clinical randomized trials are needed to confirm this result. Fourth, the lasers used in the 9 articles included in this study are not uniform, which could be a source of intergroup heterogeneity. Finally, there is significant heterogeneity in maximum urine flow rate and surgical duration, and due to the limited sample size, subgroup analysis could not be conducted. Further research is still needed to confirm these results.

However, this study addresses the limitations of previous meta-analyses. Firstly, we have included 3 new articles, involving a total of 569 patients. Moreover, the studies included in our research all had a stricture length of <2 cm. Therefore, this study aimed to integrate existing clinical research, systematically compare the superiority of laser and cold knife urethrotomy in treating urethral strictures (<2 cm) and provide the latest evidence for clinical decision-making by healthcare professionals.

## 6. Conclusions

Our study results indicate that, in the treatment of urethral strictures (<2 cm), Holmium laser urethrotomy has greater superiority in terms of long-term (12-month) Qmax, 1-year recurrence rate, and bleeding complications compared to cold knife urethrotomy. Therefore, laser urethrotomy may be a better choice for treating patients with urethral strictures at present. However, these results still need to be confirmed through further large-scale, prospective, multicenter, and long-term follow-up randomized controlled trials (RCTs).

## Author contributions

**Conceptualization:** Jiao Qin.

**Data curation:** ChongJian-Wang.

**Formal analysis:** ChongJian-Wang.

**Investigation:** Jiao Qin, Yang Liu, HongYuan-Li.

**Methodology:** Yang Liu.

**Project administration:** Zhi Wen, HongYuan-Li.

**Resources:** Zhi Wen.

**Software:** Zhi Wen.

**Supervision:** XueSong-Yang.

**Validation:** HaoTian-Huang.

**Visualization:** HaoTian-Huang.

**Writing – original draft:** CaiXia-Chen.
